# Knockin mouse models demonstrate differential contributions of synaptotagmin-1 and -2 as receptors for botulinum neurotoxins

**DOI:** 10.1371/journal.ppat.1009994

**Published:** 2021-10-18

**Authors:** Hatim Thaker, Jie Zhang, Shin-Ichiro Miyashita, Vivian Cristofaro, SunHyun Park, Ali Hashemi Gheinani, Maryrose P. Sullivan, Rosalyn M. Adam, Min Dong

**Affiliations:** 1 Department of Urology, Boston Children’s Hospital, Harvard Medical School, Boston, Massachusetts, United States of America; 2 Department of Surgery, Harvard Medical School, Boston, Massachusetts, United States of America; 3 Department of Microbiology, Harvard Medical School, Boston, Massachusetts, United States of America; 4 Faculty of Bioindustry, Tokyo University of Agriculture, Hokkaido, Japan; 5 Division of Urology, Veterans Affairs Boston Healthcare System, Boston, Massachusetts, United States of America; 6 Department of Surgery, Brigham and Women’s Hospital, Harvard Medical School, Boston, Massachusetts, United States of America; 7 R&D Center for Advanced Pharmaceuticals & Evaluation, Korea Institute of Toxicology (KIT), Daejeon, Republic of Korea; University of Pittsburgh School of Medicine, UNITED STATES

## Abstract

Botulinum neurotoxins (BoNTs) are the most potent toxins known and are also utilized to treat a wide range of disorders including muscle spasm, overactive bladder, and pain. BoNTs’ ability to target neurons determines their specificity, potency, and therapeutic efficacy. Homologous synaptic vesicle membrane proteins synaptotagmin-1 (Syt1) and synaptotagmin-2 (Syt2) have been identified as receptors for BoNT family members including BoNT/B, DC, and G, but their contributions at physiologically relevant toxin concentrations in vivo have yet to be validated and established. Here we generated two knockin mutant mouse models containing three designed point-mutations that specifically disrupt BoNT binding in endogenous Syt1 or Syt2, respectively. Utilizing digit abduction score assay by injecting toxins into the leg muscle, we found that Syt1 mutant mice showed similar sensitivity as the wild type mice, whereas Syt2 mutant mice showed reduced sensitivity to BoNT/B, DC, and G, demonstrating that Syt2 is the dominant receptor at skeletal neuromuscular junctions. We further developed an in vivo bladder injection assay for analyzing BoNT action on bladder tissues and demonstrated that Syt1 is the dominant toxin receptor in autonomic nerves controlling bladder tissues. These findings establish the critical role of protein receptors for the potency and specificity of BoNTs in vivo and demonstrate the differential contributions of Syt1 and Syt2 in two sets of clinically relevant target tissues.

## Author summary

Establishing receptors that mediate binding and entry of toxins into host cells is fundamental to understanding the specificity of toxins. The in vivo contribution of candidate receptors can be difficult to elucidate if they are critical for survival. Such a limitation exists for botulinum neurotoxins (BoNTs), a family of bacterial toxins. They are potential bioterrorism agents and are also used to treat growing numbers of medical conditions. Previous studies identified synaptotagmin-1 (Syt1) and homologous synaptotagmin-2 (Syt2) as receptors for BoNT members including type B, G, and DC. However, as Syt1 and Syt2 are essential for survival of animals, there is no viable loss-of-function animal model to establish their role in vivo. Here we created and evaluated two mutant mouse lines containing designed point-mutations in endogenous Syt1 or Syt2, respectively, that specifically disrupt toxin binding. Using these tailor-made mouse models, we demonstrated that Syt2 is the dominant receptor for BoNT/B, DC, and G at skeletal muscle neuromuscular junctions, whereas Syt1 is the dominant receptor in autonomic nerves in bladder tissues. These findings validate the role of Syt1 and Syt2 as toxin receptors in animal models and reveal their differential contributions in two clinically relevant tissues.

## Introduction

Botulinum neurotoxins (BoNTs) are a family of bacterial protein toxins that target neurons and act by blocking neurotransmitter release in nerve terminals [1–4]. They are the most potent toxins known to humans and classified as a top potential bioterrorism agent (category A and tier 1) in the United States [5].

BoNTs are composed of two chains: a light chain (LC, ~50 kDa) that is a zinc-dependent protease, and a heavy chain (HC, 100 kDa) comprising a membrane translocation domain (H_N_, ~ 50 kDa) and a receptor-binding domain (H_C_, ~50 kDa) [1–4]. The two chains are connected through a single inter-chain disulfide bond. BoNTs recognize specific receptors on nerve terminals using their H_C_ domains and enter neurons via receptor-mediated endocytosis. Acidification of endosomes then induces membrane translocation of the LC across endosomal membranes into the cytosol, where the inter-chain disulfide bond is reduced. The released LC then specifically cleaves a set of neuronal proteins required for synaptic vesicle exocytosis.

There are seven major BoNT serotypes (BoNT/A-G), produced by various anaerobic spore-forming *Clostridium* species. BoNT/A, C, and E cleave synaptosomal-associated protein of 25 kDa (SNAP-25), BoNT/B, D, F, and G cleave homologous vesicle associated membrane protein (VAMP) 1, 2, and 3, and BoNT/C also cleaves a plasma membrane protein syntaxin 1 [6–13]. Cleavage of any one of these proteins prevents synaptic vesicle exocytosis [14]. Blocking neurotransmitter release in peripheral motor nerve terminals results in flaccid muscle paralysis, causing a disease known as botulism in humans and animals [1–3]. In addition to the seven major serotypes, there are naturally-occurring mosaic toxins such as BoNT/CD, BoNT/DC, and BoNT/HA (also known as BoNT/H or BoNT/FA) [15–20] and many subtypes [21]. Recent studies also identified a number of BoNT-like toxins such as BoNT/X, BoNT/En (also known as eBoNT/J), and PMP1 [22–25], which cleave SNARE proteins as well.

As the general population is not vaccinated against BoNTs, the underlying mechanism of BoNT-induced muscle paralysis has been exploited for various therapeutic purposes [26,27]. Minute amounts of BoNTs can be injected locally to inhibit overactive nerves. BoNTs are now used to treat a growing list of medical conditions, ranging from skeletal muscle disorders such as dystonia and spasticity to overactive bladder, overactive glands, and pain. Among the seven BoNTs, BoNT/A and BoNT/B are the two serotypes approved by the FDA for clinical use.

The extraordinary specificity of BoNTs to target nerve terminals is a key factor for their potency and for their success in clinical use. It is well established that BoNTs bind to presynaptic nerve terminals via a double-receptor modality [1,28,29]. The first set of receptors are cell membrane complex gangliosides [30], which are sialic-acid-containing glycosphingolipids enriched on neuronal membranes and mediate low-affinity attachment of BoNTs to neuronal surfaces. The key role of complex gangliosides has been demonstrated in vivo using knock-out (KO) mouse models lacking essential enzymes for biosynthesis of complex gangliosides [16,31–39].

Most BoNTs also require protein receptors that mediate high-affinity binding and endocytosis into cells. Previous studies have identified the synaptic vesicle membrane protein SV2 as a receptor for BoNT/A, D, and E, and another synaptic vesicle membrane protein synaptotagmin-1 (Syt1) and homologous synaptotagmin-2 (Syt2) as receptors for BoNT/B, G, and BoNT/DC [16,34,35,37,40–46].

The toxin-binding sites have been mapped to the luminal domains of SV2 and Syt1/Syt2. Both SV2 and Syt1/Syt2 reside on synaptic vesicles, and their luminal domains are transiently exposed onto the pre-synaptic membrane during neurotransmitter release; subsequent endocytosis and recycling of these vesicle proteins mediate efficient BoNT entry into neurons. Co-crystal structures of BoNT/A, BoNT/B, and BoNT/DC in complex with their receptor fragments containing the toxin-binding sites have been resolved [43,44,47–51]. A triple complex of BoNT/B, Syt2 fragment, and complex gangliosides has been reported as well [52]. In addition, it was recently shown that Syt1/Syt2 may bind to complex gangliosides directly and together they present the high-affinity toxin binding site [53].

Despite this rapid progress in understanding toxin-receptor interactions, the contribution of SV2 and Syt in vivo at physiologically relevant toxin concentrations remains to be demonstrated. The major challenges are the existence of multiple redundant members (SV2 includes three members SV2A, SV2B, and SV2C) and inability to generate receptor-null KO mouse models: mice lacking SV2A, Syt1, or Syt2 cannot survive to adulthood [54–58]. In addition, Syt1/Syt2 and SV2 play a critical role in exocytosis/endocytosis of synaptic vesicles, which is the entry pathway for the majority of BoNTs. Therefore, any defects in Syt1/Syt2 and SV2A may reduce toxin entry into neurons indirectly.

Considering these difficulties, one approach to assess the receptor role in vivo is to generate knockin (KI) mice containing point mutations in endogenous SV2 or Syt genes that specifically disrupt toxin binding but maintain the normal function of these essential proteins. While the exact function of SV2 remains unknown, Syt1 and Syt2 are well established as Ca^2+^ sensors that control synaptic vesicle exocytosis and their functional domains are in their cytoplasmic region [57–60]. Syt1 and Syt2 contain a short luminal domain (~ 53–61 amino acids) and BoNT/B recognizes a short luminal amphipathic helix region adjacent to the single transmembrane domain of Syt1/Syt2. The identical toxin binding region is recognized by BoNT/G and BoNT/DC as well [43,44,47,61–63]. Therefore, Syt1 and Syt2 are ideal candidates for generating KI mice to evaluate their role as toxin receptors.

Specific point mutations within the toxin binding site have been designed to disrupt BoNT/B binding on cultured neurons [16,35]. Furthermore, it was found that human Syt2 contains a residue change within the toxin-binding site that reduces binding of BoNT/B, G, and BoNT/DC [16,64]. Consistent with these observations, a KI mouse line expressing Syt2 containing the entire luminal domain of human Syt2 showed reduced sensitivity to BoNT/B in vivo [65]. However, these KI mice that express the modified Syt2 showed a dominant partial male infertility [65], suggesting that replacing the entire luminal domain may still disrupt Syt2 function/stability.

To evaluate and establish the role of protein receptors for BoNTs in vivo, here we created and evaluated two novel KI mouse lines containing designed point mutations in the BoNT-binding domain of endogenous Syt1 and Syt2, respectively. Breeding these two lines also generates double KI mice with both Syt1 and Syt2 containing designed mutations. These mice showed no obvious defects including normal levels of male fertility, indicating that mutations are well tolerated. Using these three KI lines, we demonstrate conclusively that Syt1 and Syt2 are critical for the extreme potency of BoNT/B, G, and DC in vivo. We further developed a novel in vivo bladder injection assay to evaluate BoNT actions on bladder function. Using this assay and our Syt1 and Syt2 KI mice, we established that Syt1 is the dominant toxin receptor in bladder tissues, in contrast to the skeletal muscles where Syt2 is the dominant receptor.

## Results

### Characterizing triple point-mutations within the toxin-binding region

**Fig 1:**
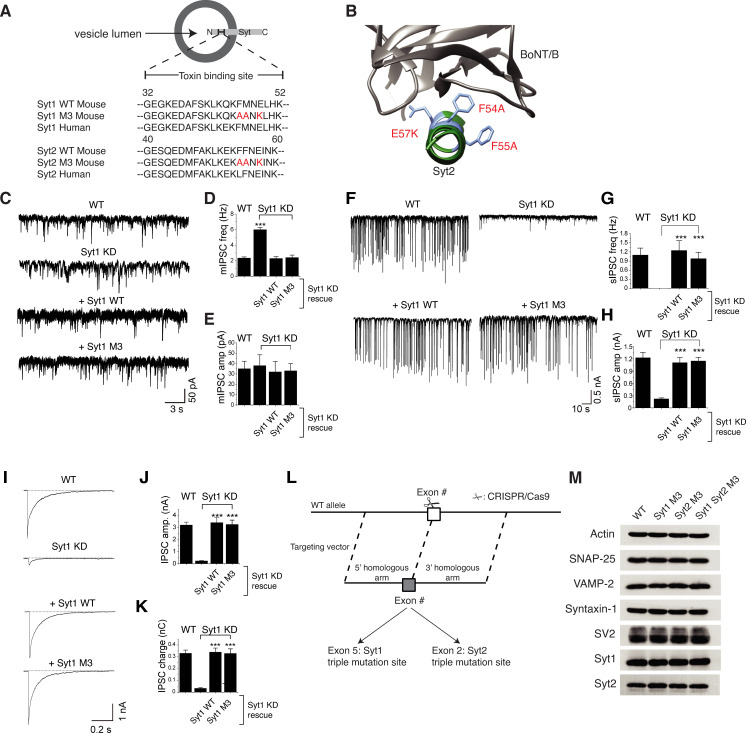
Characterizing triple point-mutations and generation of Syt1^M3^ and Syt2^M3^ KI mice. **A.** Sequence alignment of the BoNT-binding region in Syt1 and Syt2 (residues 32 to 52 in Syt1 and 40 to 60 in Syt2). Residues highlighted in red mark the designed triple mutations (Syt1^M3^ and Syt2^M3^) that abolish BoNT-binding. **B.** Co-crystal structure of the BoNT/B-Syt2 complex (PDB:4KBB) with the triple mutation sites (F54A, F55A, E57K) highlighted. **C-E.** Miniature inhibitory postsynaptic currents (mIPSC) were monitored and analyzed by whole-cell patch-clamp recording in cultured rat cortical neurons, with representative traces shown in C, frequency (freq) in D, and amplitude (amp) in E. WT: wild type neurons; Syt1 KD: neurons with their endogenous Syt1 knocked down by shRNA expressed via lentiviral transduction; Syt1^WT^: WT Syt1 is expressed in Syt1 KD neurons via lentiviral transduction; Syt1^M3^: Syt1^M3^ mutant was expressed in Syt1 KD neurons via lentiviral transduction. Syt1 KD increased mIPSC frequency, which is restored by expression of either Syt1^WT^ or Syt1^M3^. For each condition, we recorded from 12–15 neurons from a total of four coverslips. Data shown are means ± SEM. Statistical analysis was performed with Student’s t-test (***P < 0.001). **F-H.** Spontaneous inhibitory postsynaptic currents (sIPSC) were monitored and analyzed by whole-cell patch-clamp recording in cultured rat cortical neurons, with representative traces shown in F, frequency in G, and amplitude in H. The amplitude and frequency of sIPSC were greatly deceased in Syt1 KD, and both Syt1^WT^ and Syt1^M3^ restored normal levels of sIPSC. Data shown are means ± SEM. Statistical analysis was performed with Student’s t-test (***P < 0.001). **I-K.** Evoked inhibitory postsynaptic currents (IPSC) were monitored and analyzed by whole-cell patch-clamp recording in cultured rat cortical neurons, with representative traces shown in I, amplitude in J, and total charge transfer in K. The amplitude and charge of IPSC were greatly deceased in Syt1 KD, and both Syt1^WT^ and Syt1^M3^ restored normal levels of IPSC. **L.** Schematic drawing of generating Syt1 and Syt2 triple mutation KI mice via CRISPR-Cas9 approach. **M.** Expression levels of synaptic vesicle proteins Syt1, Syt2, and SV2, and toxin substrate proteins SNAP-25, VAMP-2, Syntaxin-1, analyzed by immunoblot of brain lysates, remain unchanged in Syt1^M3^ KI and Syt2^M3^ KI mice compared with WT mice.

The toxin-binding region is largely conserved between Syt1 and Syt2 (Fig 1A), and the binding interfaces between BoNT/B and Syt1/Syt2 are well-defined (Fig 1B) [43,44]. As the interface is extensive, a single point mutation may not be sufficient to disrupt the interactions. It was previously shown that Syt1 containing a triple-point-mutation at three key residues in the toxin-binding region (residues F46A, M47A, and E49K in mouse Syt1 sequence, Syt1^M3^) lost the ability to mediate binding of BoNT/B, BoNT/G, and BoNT/DC to cultured neurons [16,35], although whether these triple mutations affect other functions of Syt1 remains unknown.

To evaluate the potential impact of this triple-mutation on the function of Syt1, we examined synaptic transmission using an electrophysiological approach that can provide high temporal and kinetic resolution. A molecular replacement strategy was utilized by first knocking down (KD) endogenous Syt1 expression via lentiviral mediated shRNA expression in cultured rat cortical neurons [16], followed by expression of exogenous wild type (WT) Syt1 or Syt1^M3^ via lentiviral transduction. Whole-cell patch-clamp recordings were carried out on these cortical neurons, measuring miniature inhibitory postsynaptic currents (mIPSC), spontaneous inhibitory postsynaptic currents (sIPSC), and evoked inhibitory postsynaptic currents (IPSC).

mIPSC reflects spontaneous release of a single synaptic vesicle without stimulation. Its frequency reflects the chance of synaptic vesicle exocytosis in the presynaptic nerve terminals, and its amplitude reflects the activation of post-synaptic neurotransmitter receptors. Syt1 plays a role in clamping miniature synaptic vesicle release and Syt1 KO neurons showed increased frequency of mIPSC [60,66]. Consistently, we observed an increase of mIPSC frequency in Syt1 KD neurons (Fig 1C and 1D). Expression of either WT Syt1 or Syt1^M3^ fully restored the mIPSC frequency back to the control levels (Fig 1D and 1E).

sIPSC reflects mainly synchronized synaptic vesicle releases induced by spontaneous action potentials. Syt1 KD neurons showed greatly reduced sIPSC frequency and amplitude, which were restored by either WT Syt1 or Syt1^M3^ (Fig 1F–1H). Finally, we measured evoked IPSC by inducing synchronized synaptic vesicle exocytosis. Both Syt1 and Syt1^M3^ restored the defects in IPSC in Syt1 KD neurons (Fig 1I–1K). Together, these data demonstrate that Syt1^M3^ is indistinguishable from WT Syt1 in maintaining normal synaptic transmission.

### Syt1 and Syt2 triple-point-mutation KI mouse models

After validating that the designed triple-point-mutation does not affect the normal function of Syt1, we then generated two KI mouse lines by introducing these triple-point-mutations into endogenous Syt1 and Syt2 genes, respectively, via the CRISPR-Cas9-based method.

Briefly, the gene targeting vector containing 5’ homologous arm, target fragment (exon5 with F46A, M47A, and E49K for Syt1, and exon2 with F54A, F55A, and E57K for Syt2), and 3’ homologous arm served as a template to repair the double-strand breaks created by Cas9 with single guide RNA (sgRNA, Fig 1L). Cas9 mRNA, the template vector, and sgRNAs were co-injected into the one-cell stage fertilized mouse eggs. KI mice with endogenous Syt1 or Syt2 genes mutated were selected and are henceforth termed Syt1 M3 (Syt1^M3^) and Syt2 M3 (Syt2^M3^), respectively. Breeding these two lines together further generated a double KI line containing mutations in both Syt1 and Syt2 genes, termed Syt1 Syt2 M3 (Syt1/Syt2^M3^).

All three KI mouse lines developed normally, and no gross phenotypic differences were observed between WT and KI strains (S1 Fig). The fertility rate also appears to be normal in all three KI strains and comparable with WT mice. Immunoblot analysis of brain lysates confirmed that expression levels of Syt1, Syt2, SV2, VAMP-2, SNAP-25, and syntaxin-1 are unchanged in Syt1, Syt2, and double KI strains (Fig 1M). These data confirm that introducing the designed triple-point-mutations does not affect the normal expression/function of Syt1 and Syt2 in mice.

### Syt2 and Syt1 differentially mediate BoNT/B binding at the mouse diaphragm and bladder

**Fig 2:**
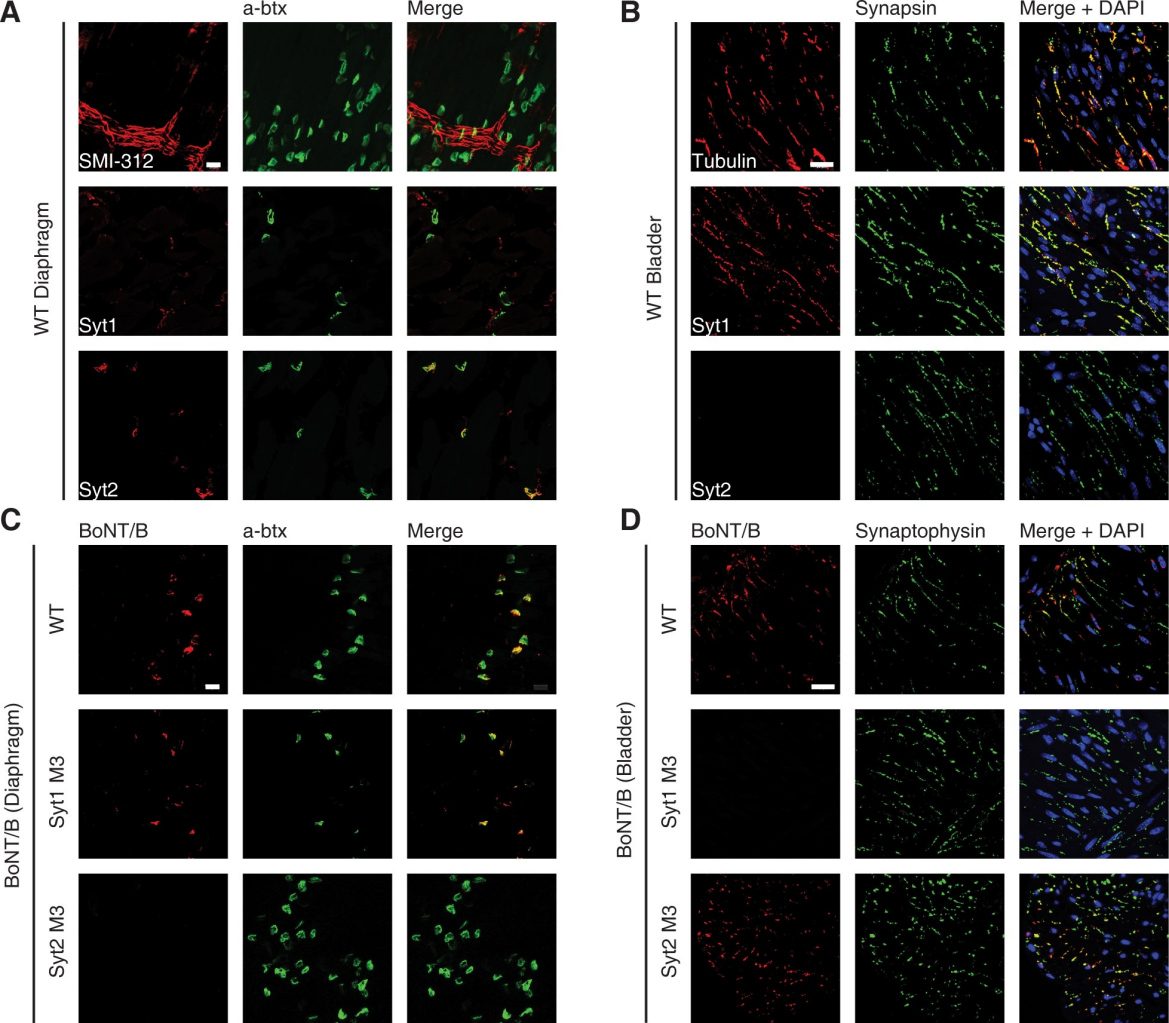
Syt2 and Syt1 are differentially expressed at the mouse diaphragm and bladder. **A.** Immunohistochemistry analysis detected expression of Syt2, but not Syt1, in motor nerve terminals at WT mouse diaphragm neuromuscular junctions (NMJs). SMI-312 antibody detects neurofilament and marks the phrenic nerve. α-Bungarotoxin (a-btx) labels post-synaptic acetylcholine receptors and serves as a marker for NMJs. Scale bar, 20 μm. **B.** Immunohistochemistry analysis of bladder sections from WT mice detected expression of Syt1, but not Syt2 in neurons. β-3 tubulin marks nerve fibers and synapsin marks the pre-synaptic site of neuronal varicosities in bladder tissues. Scale bar, 20 μm. **C.** Diaphragms isolated from WT, Syt1^M3^ KI, or Syt2^M3^ KI mice were incubated with BoNT/B (100 nM, 90 min) in high K^+^ buffer. Tissues were washed, and immunohistochemistry analysis was performed on whole mount tissues. BoNT/B binding and entry was detected on both WT and Syt1^M3^, but not Syt2^M3^ diaphragms. **D.** Bladder tissues isolated from WT, Syt1^M3^, or Syt2^M3^ mice were incubated with BoNT/B (100 nM, 90 min) in high K^+^ buffer. Tissues were washed, and immunohistochemistry analysis was performed. BoNT/B binding and entry was detected on both WT and Syt2^M3^, but not Syt1^M3^ bladders. Representative images were selected from n = 3–4 biological replicates.

To examine the in vivo role of Syt1 and Syt2, we analyzed diaphragm motor nerve terminals and bladder tissues. The former represents motor neurons controlling skeletal muscles, which are pathologically relevant targets for BoNTs. The latter represents a clinically relevant therapeutic target tissue as the toxin has been utilized to treat bladder disorders. It also presents an opportunity to understand the impact of BoNTs on the autonomic nervous system controlling smooth muscle contraction. Previous studies using immunofluorescence staining showed that all diaphragm motor nerve terminals express Syt2, with a subpopulation (~40%) expressing Syt1 in addition to Syt2 [58]. Consistently, we detected Syt2 expression by immunostaining on every neuromuscular junction (NMJ) marked by α-Bungarotoxin on isolated mouse diaphragm tissues (Fig 2A). Syt1 expression was not detectable at most motor nerve terminals examined, although low levels of signals have been observed occasionally along the axons (Fig 2A).

In the bladder, autonomic nerves are interlaced between detrusor fibers and within the lamina propria, with synapses arranged in varicosities along nerve fibers. The pan-axonal marker β-3 tubulin reveals the architecture of these nerves within the isolated mouse detrusor smooth muscle (Fig 2B). In line with previously published results [65], mouse bladders showed clear immunoreactivity to Syt1, but not Syt2, that colocalizes with the nerve terminal marker synapsin (Fig 2B). We further examined the Syt expression pattern in human bladder tissues from post-mortem specimens obtained from a commercial source. Similar to mouse tissues, human bladders displayed Syt1 immunoreactivity whereas Syt2 was not detected (S2A Fig). Consistently, RNA sequencing data, obtained from Genotype-Tissue Expression online database with 11 patients, showed much higher Syt1 mRNA levels than Syt2 in human bladder tissues (p<0.0001, S2B Fig). Similarly, mouse bladder tissues showed detectable expression of Syt1 but not Syt2 at the mRNA level(S2C Fig).

We next evaluated whether Syt1 and Syt2 mediate BoNT binding to nerve terminals in diaphragm and bladder tissues dissected from WT, Syt1^M3^, or Syt2^M3^ mice. Tissues were exposed to a BoNT/B mutant protein containing two point-mutations in its LC (R369A/Y372F) that abolish its toxicity. Bound BoNT/B was detected by immunostaining. Consistent with the differential expression patterns of Syt1 and Syt2, we found that BoNT/B can bind to diaphragm motor nerve terminals from Syt1^M3^, but not Syt2^M3^ mice (Fig 2C). In contrast, BoNT/B can bind to bladder tissues from Syt2^M3^, but not Syt1^M3^ mice (Fig 2D). These findings demonstrate that Syt2 is the dominant receptor mediating BoNT/B binding at diaphragm motor nerve terminals, and Syt1 is the dominant receptor mediating toxin binding to autonomic nerves in bladder tissues.

### BoNT/B, G, and DC showed reduced potency and toxicity on Syt2^M3^ mice

Syt1 and Syt2 KI mice for the first time offer an opportunity to evaluate the contribution of protein receptors in vivo at pathophysiological concentrations of BoNTs. To assess the potency and toxicity of BoNTs in mice, we utilized the well-established digit abduction score (DAS) assay by injecting toxins into the gastrocnemius muscle, which results in local paralysis of the hind limb and is reflected as the inability to open the paw during startle responses [67]. The degree of toe spreading can be scored from 0–4 (0 = no paralysis, 4 = complete paralysis) [67,68].

**Fig 3:**
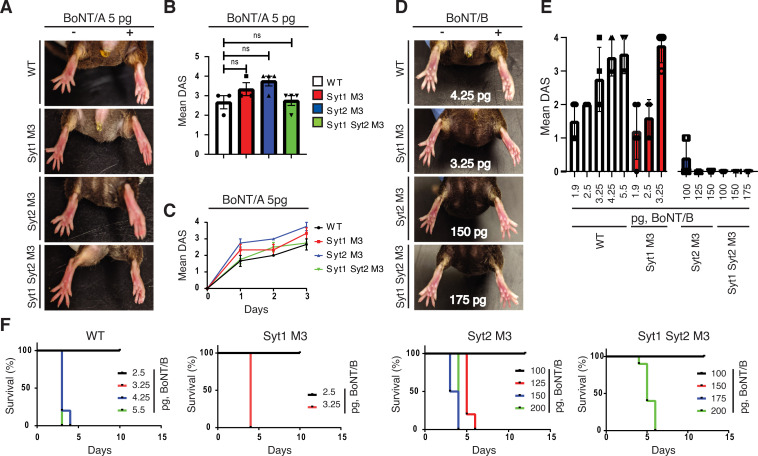
BoNT/B showed reduced potency and toxicity on Syt2^M3^ KI mice. **A-C.** BoNT/A (5 pg) was injected into the right gastrocnemius muscle of mice and muscle paralysis was scored by monitoring the degree of toe spreading in startle responses (DAS assays). BoNT/A showed the same levels of potency in inducing local muscle paralysis in DAS assays in WT, Syt1^M3^, Syt2^M3^, and Syt1/Syt2^M3^ double KI mice. Representative pictures of toe spreading are shown in A. The maximal DAS scores are plotted in B, and the mean daily DAS scores three days after toxin injection are plotted in C. Data represent mean +/- SEM. ns = not significant. **D-F.** BoNT/B showed reduced potency in inducing local paralysis and systemic toxicity in DAS assays on Syt2^M3^ KI and Syt1/Syt2^M3^ KI mice compared with WT and Syt1^M3^ KI mice. Representative images of toe spreading in DAS assays with the indicated doses are shown in D, mean DAS scores with different toxin doses are plotted in E, and the survival rates with the indicated toxin doses are plotted in F. The BoNT/B doses that resulted in moribund start at 4.25 pg for WT, 3.25 pg for Syt1^M3^, 125 pg for Syt2^M3^, and 200 pg for Syt1/Syt2^M3^ mice. Panels A, B, C–WT and Syt1^M3^ mice, n = 3, Syt2^M3^ and Syt1/Syt2^M3^ mice, n = 4. Panels D, E–all groups that received BoNT/B injection, n = 5. Except for the following groups: WT 5.5 pg BoNT/B (n = 4); Syt2^M3^ 100 pg BoNT/B (n = 10); Syt2^M3^ 150 pg BoNT/B (n = 3); Syt1/Syt2^M3^ 100 pg BoNT/B (n = 6); Syt1/Syt2^M3^ 150 pg BoNT/B (n = 6). Panel F–these numbers are from the same dataset as panels D, E.

We first examined the potency of BoNT/A as a control, which uses the same entry pathway into neurons as BoNT/B, but a different protein receptor [45,46]. Injection of BoNT/A (sub-lethal dose of 5 pg) resulted in a DAS score of 3–4 by day 3 after injection in WT mice. Syt1^M3^, Syt2^M3^, and double KI mice all showed the same levels of paralysis as WT mice (Fig 3A). The mean DAS scores and the paralysis onset time remained unchanged in all three KI mice (Fig 3B and 3C), demonstrating that these KI mice have no detectable defects in mediating functional entry of BoNT/A.

Injection of BoNT/B elicited dose-dependent DAS scores, which reach 3–4 at 4.25 to 5.5 pg by day 2–3 after injection in WT mice (Figs 3D and 3E and S3). At 4.25 and 5.5 pg, the mice became moribund after 3 days and were euthanized (Fig 3F), indicating that BoNT/B at these doses has diffused systemically away from the hindleg. Injection of BoNT/B into Syt1^M3^ mice showed a dose-dependent increase of DAS scores similar to WT mice (Figs 3E and S3). The dose that induced a score of 3–4 appears to be slightly lower in Syt1^M3^ than in WT mice (3.25 pg in Syt1^M3^ versus 4.25 in WT), and doses beyond 3.25 pg were lethal to Syt1^M3^ mice (Fig 3F and Table 1). Therefore, Syt1^M3^ mice appear to be slightly more sensitive to BoNT/B than WT mice.

**Table 1:** A list of toxin doses correlating with systemic toxicity in WT and KI mice. The full ranges of titration toxin doses and survival rates are shown in Figs 3F, 4B and 4D.

Toxin	Mouse strain	Highest administered dose resulting in 100% survival	Lowest administered dose that induces 100% death
BoNT/B	WT	3.25 pg (n = 5)	4.25 pg (n = 5)
	Syt1^M3^	2.5 pg (n = 5)	3.25 pg (n = 5)
	Syt2^M3^	100 pg (n = 10)	125 pg (n = 5)
	Syt1/Syt2^M3^	175 pg (n = 10)	200 pg (n = 4)
BoNT/DC	WT	20.5 pg (n = 5)	25.6 pg (n = 5)
	Syt1^M3^	16.4 pg (n = 5)	20.5 pg (n = 5)
	Syt2^M3^	225 pg (n = 5)	425 pg (n = 5)
	Syt1/Syt2^M3^	250 pg (n = 5)	500 pg (n = 3)
BoNT/G	WT	1.5 ng (n = 5)	3 ng (n = 5)
	Syt1^M3^	1.5 ng (n = 5)	3 ng (n = 5)
	Syt2^M3^	50 ng (n = 5)	100 ng (n = 5)
	Syt1/Syt2^M3^	50 ng (n = 5)	100 ng (n = 5)

Syt2^M3^ mice, however, showed no paralysis at all even with injection of 150 pg BoNT/B (Fig 3D and 3E). Some mice showed moribund state with 125 pg and 150 pg, limiting the dose ranges that can be assessed. The minimal dose that caused no morbidity in Syt2^M3^ mice is 100 pg (Fig 3F and Table 1). Syt1/Syt2^M3^ mice appear to be even less sensitive to BoNT/B, showing no paralysis and no morbidity with 175 pg of BoNT/B (Fig 3D–3F). Further increasing the dose to 200 pg resulted in morbidity. Considering that the moribund dose in WT mice is ~4.25 pg for BoNT/B, there is a ~47-fold reduction in toxicity in vivo for BoNT/B in Syt1/Syt2^M3^ mice.

**Fig 4:**
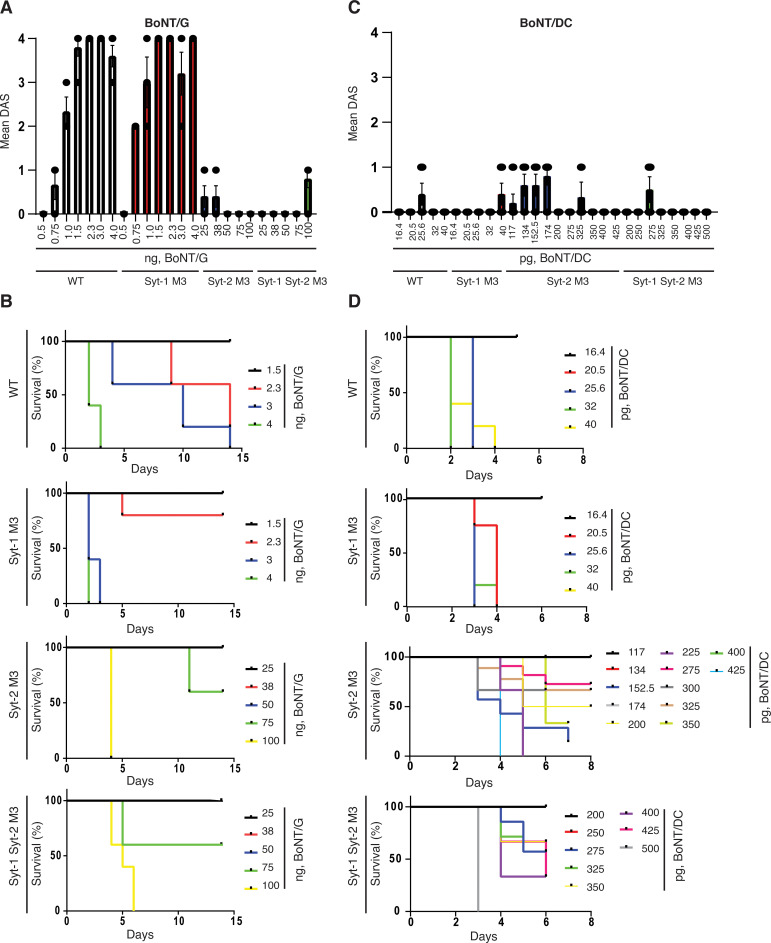
BoNT/G and BoNT/DC showed reduced potency and toxicity on Syt2^M3^ KI mice. **A-B.** BoNT/G showed reduced potency in inducing local muscle paralysis in DAS assays in Syt2^M3^ and Syt1/Syt2^M3^ mice (A), as well as reduced toxicity in systemic lethality levels (B), compared with WT and Syt1^M3^ mice. The BoNT/G doses that induced moribund are 2.3 ng in both WT and Syt1^M3^ mice, and 75 ng in Syt2^M3^ and Syt1/Syt2^M3^ mice. **C-D.** BoNT/DC did not elicit significant local paralysis before it caused death of mice in DAS assays. It showed reduced toxicity in Syt2^M3^ and Syt1/Syt2^M3^ mice compared with WT and Syt1^M3^ mice. The BoNT/DC doses that induced moribund are at 25.6 pg in WT mice, 20.5 pg in Syt1^M3^ mice, and 275 pg in both Syt2^M3^ and Syt1/Syt2^M3^ mice. Panels A and B, n = 5; panels C and D, n = 5, except for the following: Syt2^M3^, BoNT/DC 200 pg (n = 4); Syt2^M3^, BoNT/DC 275 pg (n = 4); Syt2^M3^, BoNT/DC 325 pg (n = 3); Syt2^M3^, BoNT/DC 425 pg (n = 3); Syt1/Syt2^M3^, BoNT/DC 200 pg (n = 4); Syt1/Syt2^M3^, BoNT/DC 250 pg (n = 4); Syt1/Syt2^M3^, BoNT/DC 275 pg (n = 4); Syt1/Syt2^M3^, BoNT/DC 325 pg (n = 4); Syt1/Syt2^M3^, BoNT/DC 400 pg (n = 4); Syt1/Syt2^M3^, BoNT/DC 500 pg (n = 3).

We also examined two other BoNTs, BoNT/G and BoNT/DC, that use Syt1 and Syt2 as receptors. For BoNT/G, WT and Syt1 KI mice showed similar levels of dose-dependent paralysis in DAS assays and the dose resulting in morbidity is the same for WT and Syt1^M3^ mice (Figs 4A and 4B and S3 and Table 1). Syt2^M3^ and Syt1/Syt2^M3^ mice showed greatly reduced sensitivity to BoNT/G and no paralysis at the dose that already rendered mice moribund (Figs 4A and S3 and Table 1). The dose leading to morbidity showed ~33-fold differences between Syt1/Syt2^M3^ and WT mice.

Unlike BoNT/B and BoNT/G, BoNT/DC injection led to a moribund state before developing any consistent DAS scores, indicating that this toxin is prone to diffuse away from the injection site (Figs 4C and 4D and S3 and Table 1). The highest dose that did not result in morbidity is at 20.5 pg for WT, 16.4 pg for Syt1^M3^, 225 pg for Syt2^M3^, and 250 pg for the Syt1/Syt2^M3^ mice. The doses rendering 100% of mice moribund were at 25.6 pg for WT, 20.5 pg for Syt1^M3^, 425 pg for Syt2^M3^, and 500 pg for Syt1/Syt2^M3^ mice (Table 1). These data suggest that Syt1^M3^ mice are slightly more sensitive than WT mice, whereas Syt2^M3^ and Syt1/Syt2^M3^ mice showed a 10–19-fold reduction in sensitivity to BoNT/DC compared with WT mice.

### Syt1^M3^ bladder strips are less sensitive to BoNT/B

**Fig 5:**
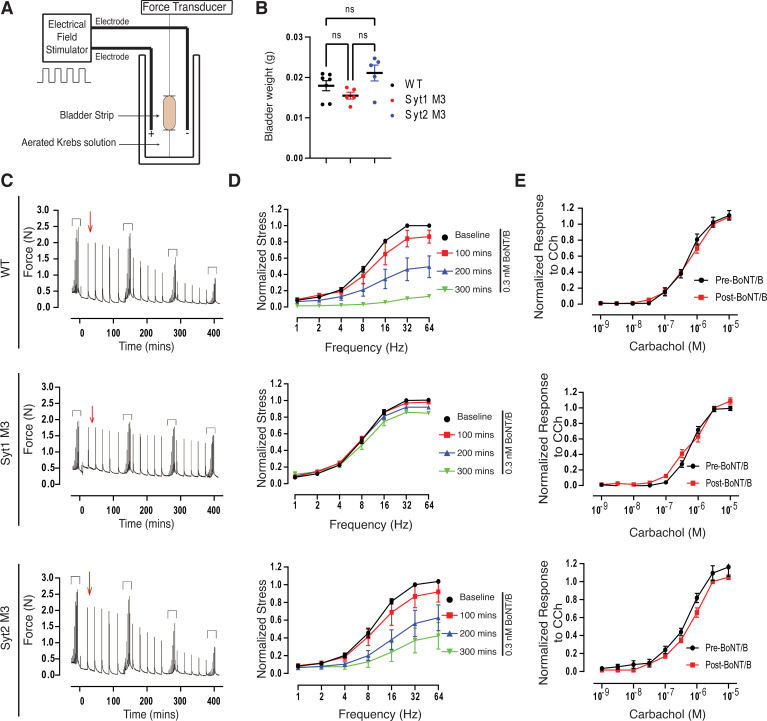
Syt1^M3^ bladder strips are less sensitive to BoNT/B in e*x vivo* bladder contraction assays. **A.** Schematic diagram of *ex vivo* bladder strip contraction assay. Bladder strips were immersed in aerated Krebs solution. Exogenous pharmacologic agents (i.e., carbachol, BoNT/B) were added directly to the solution. Trains of voltage delivered over a range of frequencies were generated by the electrical field stimulator to trigger bladder contraction, which is detected and measured by the force transducer. **B.** Bladder weights across WT and KI mice were similar prior to *ex vivo* contraction assay. ns = not significant (ANOVA). **C-D.** The nerve-evoked contraction forces of WT (n = 3), Syt1^M3^ (n = 5), and Syt2^M3^ (n = 4) bladder strips were recorded, with the representative force trace shown in C. A set of frequency-dependent stimulations (enclosed by brackets above tracing) were delivered every 100 min. After a baseline frequency-response, evoked responses at constant frequency (16 Hz) were produced every 15 min. BoNT/B (0.3 nM) was administered at red arrow. The time dependent effects of BoNT/B on nerve evoked responses are shown in frequency-response curves that were plotted in 100 min intervals, shown in D. Incubation with BoNT/B reduced contractions of WT and Syt2^M3^ bladders, but did not affect Syt1^M3^ bladders. *p<0.05, repeated measures ANOVA. **E.** Carbachol induces direct muscle contraction independent of neurotransmission. Carbachol does-response curves were generated at the beginning and end of the experiment as a control showing that bladder strips maintained their viability and contractility after incubation with BoNT/B for 300 min. p>0.05, RM ANOVA.

To analyze the role of Syt1 and Syt2 in mediating functional entry of BoNT/B into autonomic nerve terminals, we first assessed the effect of BoNT/B in paralyzing ex vivo bladder detrusor smooth muscle strips isolated from WT, Syt1^M3^, and Syt2^M3^ mice. Electrical field stimulation (EFS) induces nerve-evoked contraction of bladder strips and the contraction force can be recorded (Fig 5A) [69,70]. Bladder weights from WT, Syt1^M3^, and Syt2^M3^ mice were similar (Fig 5B), and all data were normalized to the cross-sectional area of the bladder strip. Using EFS with varying frequencies (1–64 Hz), WT and Syt2^M3^ bladder strips showed a reduction in nerve-evoked contractions after exposure to 0.3 nM BoNT/B and the contraction was almost completely inhibited after 300 min incubation (Fig 5C and 5D). In contrast, Syt1^M3^ bladders were resistant to 0.3 nM BoNT/B, showing no reduction in nerve-evoked contractions after 300 min incubation (Fig 5C and 5D). Exposure to 1 nM BoNT/B resulted in similar results, with Syt1 KI bladders showing a slight decrease of contraction after 300 min incubation (S4A Fig).

Carbachol is a post-synaptic cholinergic agonist that can directly depolarize muscles without nerve-evoked stimulation. Treatment with carbachol confirmed that both WT and KI bladder strips maintained the same level of force generation pre- and post-BoNT/B exposure (Figs 5E and S4B), demonstrating that the diminished contraction after BoNT/B exposure results from action at the pre-synaptic site and is not from reduced activation of the contractile apparatus.

### An in vivo model analyzing BoNT-induced urinary retention

**Fig 6:**
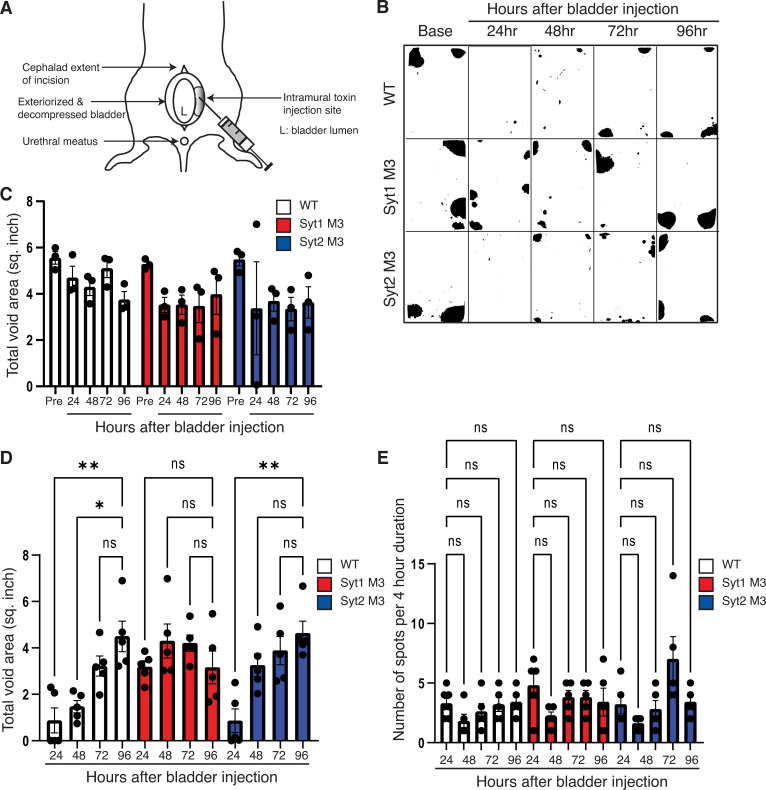
Analyzing BoNT-induced urinary retention in vivo. **A.** Schematic illustration of the bladder injection model. Mice were anesthetized with isoflurane and a low midline laparotomy was made. The bladder was exteriorized and decompressed of urine to thicken the bladder wall. BoNT/B was injected into the lateral walls of the bladder bilaterally. **B.** Mice injected with BoNT/B were subjected to 4-hour nighttime voiding spot assay every 24 hours for 4 days. Representative images for urine spots (black areas) are shown. **C.** Voiding spots quantified into total void areas (inch^2^) demonstrate similar pre-injection urine volumes across WT, Syt1^M3^, and Syt2^M3^ mice (n = 5 for each group). All groups showed a decline in voided volumes in sham controls that went through the surgery and injection procedures without toxins, likely from surgical manipulation of the bladder and the use of post-operative pain control narcotics. n = 3 for each group. **D.** At 24–48 hours post-injection, WT and Syt2 KI mice had significantly diminished voided volumes compared to Syt1 KI mice, with progressive recovery over 96 hours. *P<0.05, **P<0.005, ns = not significant. **E.** Number of voiding spots per 4-hour nighttime evaluation showed no difference across genotypes, and across pre- and post-injection time points, demonstrating that voiding frequency is unchanged across groups. ns = not significant.

To further examine the effects of BoNTs on bladder function in vivo, we developed a bladder injection assay (Fig 6A). The bladder was surgically exposed in anesthetized mice, decompressed with manual expression, and the wall injected with a sub-lethal dose of BoNT/B (3.25 pg in 2.5 μL per lateral wall, S4C Fig). The incision was then closed, and mice were allowed to recover from the surgery. We then monitored and recorded their spontaneous night-time 4-hour urinary voiding patterns using the well-established voiding spot assay by putting Whatman papers on the bottom of the cage (Fig 6B) [71].

Each mouse underwent voiding evaluation prior to toxin injection, and for four nights post-injection. There was no difference between WT and KI mice prior to toxin injection: they urinated in the corners and along the edges of the cage with large volumes, reflecting complete single-void bladder emptying (Fig 6B). The total void areas were similar among WT, Syt1^M3^, and Syt2^M3^ mice before the surgery and injection (Fig 6C). There were general declines in total void areas, which persisted for over 96 hours, in all sham controls (mice went through the surgery and injection procedure without toxins) across WT, Syt1^M3^, and Syt2^M3^ mice, which is possibly due to the impact from surgical manipulation and/or use of post-operative pain control narcotics (Fig 6C). Nevertheless, twenty-four hours after BoNT/B injection, there was a drastic reduction in total void area compared with sham controls in WT mice (Fig 6D). The void areas increased at the 48-hour compared with the 24-hour time point. The difference between 72 and 96 hours were no longer significant and similar to sham controls (Fig 6D). The urinary frequency remained unchanged from 24 to 96 hours, confirming that the voiding defect was due to urinary retention (Fig 6E). Syt2^M3^ mice showed similar results as WT mice (Fig 6D). In contrast, Syt1^M3^ mice showed no reduction in total voiding area or frequency at the 24-hour compared with 96-hour time point, demonstrating that Syt1^M3^ mouse bladders are resistant to BoNT/B in vivo at physiologically relevant toxin concentrations.

## Discussion

Here we generated KI mice with endogenous Syt1 and Syt2 mutated, containing point mutations specifically designed to disrupt BoNT binding. These mice displayed no abnormal phenotype, growth delay, or fertility issues, suggesting that the normal expression and function of Syt1 and Syt2 are maintained in KI mice. These tailor-made mouse models are ideal for the BoNT research community to analyze the contribution of Syt1 and Syt2 for BoNT/B, G, and DC at physiologically relevant toxin concentrations in vivo. They are also valuable models for the neuroscience community to investigate the differential expression/function of Syt1 versus Syt2 and could provide a way to acutely and selectively block Syt1 or Syt2 mediated neurotransmission in vivo utilizing BoNTs.

Using these KI mouse models, our studies conclusively demonstrated the key role of protein receptors for the extreme potency and toxicity of BoNTs in vivo at physiologically relevant toxin concentrations. These mouse models also allow us to distinguish the contribution of Syt1 versus Syt2 in vivo at skeletal muscle NMJs and in bladder tissues at functional levels. Syt1 and Syt2 are highly homologous to each other and can be functionally redundant. They often show distinct and complementary expression patterns across different tissues, with Syt1 as the dominant form in the forebrain areas and Syt2 as the dominant one in caudal areas like spinal cord, brainstem, and cerebellum [57,58,72,73]. In the peripheral nervous system, a previous study has detected Syt2 in all diaphragm motor nerve terminals [58]. Mutations in Syt2 gene in humans are also associated with neuromuscular phenotypes, suggesting that Syt2 plays a critical role in NMJs [74]. The role of Syt1 at NMJs is less clear. It was detected in ~40% of motor nerve terminals in a previous study [58], but we were not able to detect Syt1 at diaphragm motor nerve terminals by immunostaining. This variation may reflect the limitation in detecting proteins with low expression levels and in distinguishing closely related homologous proteins. Our finding that sensitivity of Syt1/Syt2^M3^ mice to BoNT/B is further reduced by <1.75-fold compared to Syt2^M3^ mice suggest that Syt1 is likely expressed in NMJs at low levels or in a subpopulation of neurons. However, as Syt1^M3^ mice showed similar or even a slightly enhanced sensitivity to BoNTs compared with WT mice, Syt2 is clearly the predominant and physiologically relevant receptor for BoNT/B, G, and DC at NMJs and Syt1 does not contribute meaningfully to toxin entry to skeletal motor neurons in the presence of normal levels of Syt2.

It is interesting that Syt1^M3^ showed a slightly higher sensitivity to toxins. The underlying cause for this observation remains to be elucidated and could be multifaceted. There is a possibility that reduced toxin binding to Syt1 may focus toxins toward Syt2-expressing motor neurons. Another interesting observation is that BoNT/DC showed higher tendency for systemic toxicity than both BoNT/B and BoNT/G in DAS assays. Similar results have been previously reported [75], suggesting that BoNT/DC is more likely to diffuse away from the local injection site than BoNT/B and BoNT/G. BoNT/DC showed much lower binding affinity toward rat and human Syt1 and Syt2 compared with BoNT/B and BoNT/G, which may lower its efficacy in targeting and fixation onto local motor nerve terminals [62].

Composed of smooth muscle fibers controlled by autonomic nerves, the bladder was immunoreactive for Syt1, not Syt2. To define this difference further, Syt1^M3^ mice were treated with BoNT/B, resulting in no binding, implicating Syt1 as functionally important for BoNT/B binding in smooth muscle tissues. To further explore this finding, *ex vivo* organ bath studies showed that isolated Syt1^M3^ detrusor strips were less susceptible to BoNT/B, whereas Syt2^M3^ bladder strips showed a sensitivity similar to WT bladder strips.

To further evaluate the role of Syt1 versus Syt2 in bladder tissues in vivo, we injected BoNTs into the bladder wall and then measured urinary retention using the well-established voiding spot assays. BoNTs are widely used to treat urological disorders. However, previous evaluation of BoNT effects on bladders in vivo are largely limited to detecting cleavage of toxin substrates such as SNAP-25 [76]. Our study established a method to analyze the impact of BoNTs on bladder functions at physiologically relevant toxin concentrations in vivo. Using this model, we found that bladders in Syt1^M3^ mice are less sensitive to BoNT/B, whereas bladders in Syt2^M3^ mice showed similar levels of sensitivity as the bladders in WT mice. These findings demonstrated that Syt1 is the dominant receptor in bladder tissues in vivo at physiologically relevant toxin concentrations.

The clinical use of BoNT/B for treating muscle paralysis such as dystonia showed a higher chance of side effects such as dry mouth compared with BoNT/A, which is likely due to its impact on the autonomic nervous system [77]. The differential expression of Syt1 and Syt2 means that tailor-made, site-specific BoNTs can be potentially developed. For instance, an engineered BoNT/B with enhanced binding to Syt2 and reduced binding to Syt1 may reduce its side effects on autonomic nerves.

On the other hand, BoNT/B showed higher efficacy for targeting bladder tissues than commonly used BoNT/A [65,78]. Thus, an engineered BoNT/B or a BoNT/AB chimeric toxin utilizing the LC-H_N_ of BoNT/A and BoNT/B-H_C_ could provide a better choice than BoNT/A to target the autonomic nervous system. An engineered BoNT/B with enhanced binding to Syt1 and reduced binding to Syt2 might be able to target bladder tissues and autonomic nerves more efficiently and reduce the unwanted paralysis of skeletal muscles. BoNT/B naturally showed reduced binding to human Syt2 due to a single residue change (F54L) [16,64]. By combining structure-based rational design and saturation mutagenesis screening, we have previously identified mutations in BoNT/B that can restore binding of BoNT/B to human Syt2 [79]. Similar approaches may be pursued to create Syt1 or Syt2-selective BoNT/B tailored toward different therapeutic uses with enhanced specificity, efficacy, safety, and reduced side effects.

## Materials and methods

### Ethics statement

All animal studies were conducted at Boston Children’s Hospital and approved under Institutional Animal Care and Use Committee (protocol numbers 18-10-3794R and 19-12-4065). Studies were performed in accordance with the guidelines of the Association for Assessment and Accreditation of Laboratory Animal Care International. Boston Children’s Hospital institutional animal care program holds an active animal welfare assurance on file with the Office of Laboratory Animal Welfare at the National institutes of Health, which describes the hospital adherence to Public Health Service’s Policy on Humane Care and Use of Laboratory Animals.

### Biosafety

Procedures were approved by the Institute of Biosafety Committee at Boston Children’s Hospital (IBC-P00000501). Active BoNTs are purchased from Metabiologics (Madison, Wisconsin, USA) and stored in a locked freezer, and toxin laden reagents, tips, needles, and other consumables are exposed to 10% bleach for decontamination prior to disposal.

### Neuron culture and viral transfection

The cortexes were dissected from the brain of E18-19 rat embryos, and dissociated by papain following the manufacturer’s instructions (Worthington Biochemical, NJ). Neurons were plated at 100,000 cells/cm^2^ on circular glass coverslips coated with poly-D-lysine (Sigma-Aldrich). Cells were cultured in Neurobasal medium supplemented with B-27 (2%) and Glutamax (Invitrogen). Transduction of neurons was carried out using Lentivirus. This vector contains two separate neuronal-specific synapsin promoters. A Lox-Syn-Syn lentivirus vector was used for all lentiviral constructs in neurons as described previously [37]. A Lox-U6-Syn lentiviral vector was generated by replacing one synapsin promoter in Lox-Syn-Syn with a U6 promoter. A Syt1 shRNA KD construct was generated using the following primers: 5’-GATCCGTGCAAGTGGTGGTAACTGGCTCGAGGCAGTTACCACCACTTGCACTTTTTTA-3’, targeting base pairs 1063–1081 of rat Syt1. KD-resistant Syt1 was generated by changing GTGGTGGTA to GTAGTAGTG within the targeting site. Lentiviruses were added to neurons at DIV6 (days in vitro). Experiments were carried out generally using DIV (days in vitro) 14–18 neurons.

### Electrophysiology

Whole-cell patch-clamp recordings were made from dissociated cortical cultures. All recordings were made 14–18 days after primary neurons were plated on coverslips. The internal solution for whole-cell experiments contained the following (in mM): 135 CsCl, 10 HEPES, 1 EGTA, 1 Na-GTP, 4 Mg-ATP and 10 QX-314 (pH 7.4, adjusted with CsOH). The resistance of pipettes filled with intracellular solution varied between 3 and 5 MΩ. After formation of the whole-cell configuration and equilibration of the intracellular pipette solution, the series resistance was adjusted to 10–15 MΩ. Cells with initial Rs exceeding 20 MΩ were excluded from analysis. Synaptic currents were monitored with an EPC-10/2 amplifier (HEKA) at -70 mV holding potential. Presynaptic inputs were stimulated with a bipolar electrode (FHC; www.fh-co.com; Cat# CBAEC75-Concentric Bipolar Electrode OP: 125m SS; IP: 25m Pt/l) that was placed nearby the patched neuron and used to apply a 0.15 mA, 1 ms current injection. The distance between the tip of the stimulation electrode and the cell body of the postsynaptic neuron varied between 100 and 150 μm. The frequency, duration, and magnitude of the extracellular stimulus were controlled with a Stimulus Generator 4002 (Multichannel systems) synchronized with Patch Master stimulation/acquisition software. The bath solution contained the following (in mM): 140 NaCl, 5 KCl, 2 CaCl2, 1 MgCl2, 10 HEPES, 10 glucose (pH 7.4, adjusted with NaOH). Spontaneous inhibitory postsynaptic currents (sIPSCs) and evoked inhibitory postsynaptic currents (IPSCs) were pharmacologically isolated by adding AMPA and NMDA receptor blockers CNQX (20 μM) and APV (50 μM) to the extracellular bath solution. Spontaneous miniature inhibitory postsynaptic currents (mIPSCs) were monitored in the presence of 1 μM tetrodotoxin (TTX) to block action potentials. All experiments were performed at room temperature (RT). Data were analyzed using Clampfit 10 (Molecular Devices), Origin8 software (Mocrocal Inc.), MiniAnalysis software (Synaptosoft), and Igor (Wavemetrics). Statistical analysis was performed with Student’s t-test (*P < 0.01). All data shown are means ± SEM.

### Immunofluorescence staining

Bladder tissues were dissected and washed with PBS. For BoNT staining, the bladders were incubated in 100 μM of BoNT/B_RY_ (mutant protein with two point-mutations in its LC (R369A/Y372F) that abolish its toxicity) in high K^+^ buffer. Tissues were fixed in 10% formalin overnight, embedded in paraffin, and sectioned (10 μm thick). Sections were incubated with primary antibodies at 4° C overnight (anti-BoNT/B, anti-synapsin, anti-synaptophysin, anti-Syt1, and anti-Syt2). After washing with PBS, tissues were incubated with secondary antibodies for 60 minutes at RT and counterstained with DAPI (Vector Labs). Images were captured via Zeiss LSM 810 confocal microscope and processed using FIJI (only brightness and contrast was adjusted). Human bladder sections were purchased from Biochain (Cat#T2234010) and subjected to immunofluorescence analysis in a similar fashion.

Whole diaphragm tissues were harvested and fixed with 2% paraformaldehyde for 3 hours at 4° C, followed by washes with 0.1% Triton-X100-PBS. For BoNT/B staining, diaphragms were incubated in 100 μM of BoNT/B_RY_ in high K^+^ buffer for 90 minutes before fixation. Tissues were then incubated in 0.1 M glycine-PBS for 60 minutes, then in blocking buffer (2% BSA, 4% normal goat serum in 0.5% Triton-X100-PBS) for 60 minutes. Primary antibodies against BoNT/B and α-Bungarotoxin were applied (S1 Table). Whole tissues were mounted with DAPI and imaged via Zeiss LSM 810 confocal microscope.

### Generation of Syt1 and Syt2 KI mice

Syt1^M3^ and Syt2^M3^ mice were generated by CRISPR/Cas9 based approach. For Syt1^M3^ mice, the Phe46Ala, Met47Ala, and Glu49Lys triple mutations were introduced in exon 5. For Syt2^M3^ mice, the Phe54Ala, Phe55Ala, and Glu57Lys triple mutations were introduced in exon 2 using an overlap extension-PCR method. Briefly, sgRNAs were designed by CRISPR design tool (http://crispr.mit.edu/) to target either a region upstream or downstream of the exon 5 of Syt1 or exon 2 of Syt2, and then were screened for on-target activity using a Universal CRISPR Activity Assay (UCA^TM^, Biocytogen Pharmaceuticals(Beijing)Co., Ltd). For Syt1, the two sgRNAs were sgRNA-1: AGTCTATAATAGCATAATACTGG and sgRNA-2:GAGCACGAAGTCTAACCCGTGGG. For Syt2, one sgRNA was used (sgRNA-1: GAAGAATTTCTCCTTCAGCTTGG). The gene targeting vectors contain 5’ homologous arm, target fragment (exon5 with Phe46Ala, Met47Ala & Glu49Lys triple mutations; exon2 with Phe54Ala, Phe55Ala & Glu57Lys triple mutations) and 3’ homologous arm. Cas9 mRNA, targeting vector and sgRNAs were co-injected into the cytoplasm of one-cell stage fertilized C57BL/6 eggs. The injected zygotes were transferred into oviducts of Kunming pseudo-pregnant females to generate F0 mice. F0 mice with expected genotype confirmed by tail genomic DNA PCR and sequencing were mated with C57BL/6 mice to establish germline-transmitted F1 heterozygous mice. F1 heterozygous mice were genotyped by tail genomic PCR, southern blot and DNA sequencing.

### Characterization of KI mice

Examination of transgenic and non-transgenic siblings was conducted weekly via body weight, and progeny counts. Additionally, WT and KI mouse brain detergent extracts were prepared by homogenization of three fresh adult brains per strain, in a solution of 10 mM Tris (pH 7.5), 0.5% SDS, 1% Triton X-100, 0.75 mM NaCl, 20 mM SDC and protease inhibitor (APE Bio K1008). Homogenized tissue was centrifuged at 14,000 rpm for 20 minutes at 4° C. Supernatants were collected, and 50 μg of protein was subjected to SDS-PAGE in Tris-HEPES running buffer at 120 V for 30–40 minutes. After transfer onto nitrocellulose membranes, the blots were blocked in 5% dehydrated non-fat milk in 0.1% TBST for 60 minutes at RT, then probed with antibodies directed against Syt1, Syt2, and other synaptic proteins (listed in S1 Table) for 60 minutes. After species-specific HRP-conjugated antibody (Sigma-Aldrich) incubation (60 minutes at RT), immunoblot analysis was conducted using enhanced chemiluminescence method (Pierce).

### Animal handling

For *in vivo* mouse digit abduction score (DAS) assay, male and female WT and Syt1^M3^, Syt2^M3^, and Syt1/Syt2^M3^ mice, aged 8–14 weeks and weighing 18–25 g were utilized. For mouse bladder *in vivo* injections and *ex vivo* contraction assay, female mice were used (age 8–10 weeks, weighing 18-22g). We used only female mice for bladder toxin injection assays because there was a potential risk of complete urinary retention from bladder paralysis after BoNT/B injection. If complete urinary retention did occur, our protocol requires manual bladder expressions to evacuate urine every 12 hours. Manual bladder expression is difficult in adult male mice. All mice were housed with five mice per cage, with free access to water and pelleted diet ad libitum.

### Mouse digit abduction score (DAS) assay

BoNTs were diluted in 0.2% gelatin-phosphate buffer (pH 6.3). WT and transgenic mice were labeled by a tail marking, and after brief isoflurane anesthesia, 5 μL of diluted toxin was injected into the right gastrocnemius muscle with a Hamilton syringe (30G needle). Each cohort included 3–11 mice/dose, and mice were assigned to a dose in a randomized manner. Daily weights were obtained along with the score of digit abduction (0; normal, to 4; maximal paralysis). Mice were monitored until expiration, or recovery from paralysis. Determination of a moribund state is conducted using a previously reported systemic toxicity symptom scoring table [68]. A clinical score is given to any mouse displaying signs and symptoms of systemic toxicity. Any mouse exhibiting a score of >5 is considered moribund and euthanized [68].

### Mouse detrusor strip ex vivo contractility studies

Bladders from WT and transgenic mice were harvested, excluding the ureteral orifices, and placed in ice-cold Krebs buffer (NaCl 120 mM, KCl 5.9 mM, NaHCO_3_ 25 mM, Na_2_H_2_PO_4_ 1.2 mM, MgCl_6_H_2_O 1.2 mM, CaCl_2_ 2.5 mM, dextrose 11.5 mM). Each bladder was cut into three longitudinal strips and mounted in tissue chambers at 37° C with continuous bubbled mixture of 95% O_2_ and 5% CO_2_. Bladder strips were attached to a force transducer (Grass Instruments) and stretched to 0.5 g of passive force. After an equilibration period of 60 minutes, contractile responses to carbachol (1–10 μM) were performed. Once the tissues recovered, electrical field stimulation (EFS; 1-64Hz, 30V 0.5 ms pulse width, 10 second duration) was conducted. Thereafter, 0.3 nM or 1 nM BoNT/B was added to the tissue chamber. At 100, 200, and 300 minutes, a frequency-response study was conducted, with repeated 16 Hz stimulation every 15 minutes in between. A carbachol stimulation response was then done prior to study termination. Data are expressed as force (mN, millinewtons, converted from grams) normalized by tissue cross-sectional area.

### Direct bladder injections and spontaneous voiding spot assay

Female mice were deeply anesthetized using 2–3% isoflurane, and a low midline laparotomy was performed. The bladder was exteriorized, decompressed with manual expression to thicken the bladder wall, and BoNT/B was injected (2.5 μL per lateral wall) using a 35G Hamilton needle. The incision was closed with absorbable suture (5–0 Vicryl, Ethicon). Post-operative care included daily 0.9% normal saline and enrofloxacin injections, to prevent urinary tract infection, and monitoring every 12 hours. Analgesia was provided using buprenorphine sustained release once post-operatively.

Forty-eight hours prior to injection, individual mice were placed in a rectangular polycarbonate cage (without wire flooring), lined with Whatman paper on the bottom (Cat #3030–917), from 8pm to 12am; this served as the baseline voiding pattern for each mouse. Mice were fed ad libitum, but water was restricted to avoid water dripping onto the filter paper. Thereafter, on post-operative night 1–4, the voiding spot assay was repeated for each BoNT/B injected mouse. Mice were returned to their communal cages after the 4-hour period. Papers were dried, imaged via ChemiDoc MP imaging system (UV trans illumination, 0.7 sec exposure time) and processed via a FIJI macro. Each image was converted to 8-bit, and thresholded by dark and light void areas, prior to conversion to a binary image. Regions of interest, or void areas, were then overlaid onto the original images to perform quantification.

### GTEx RNA-seq data acquisition from The Human Protein Atlas

Human urinary bladder specific expression of Syt1, Syt2, syntaxin-1 and synaptophysin was accessed via http://www.proteinatlas.org, which aggregates the Genotype-Tissue Expression (GTEx, Broad Institute of MIT and Harvard) project data. RNA-sequencing data from human urinary bladder was mapped based on RSEMv1.2.22 (v7) and the transcripts per million (TPM) values from 11 patients (5 female, 6 male) were extracted from the specific gene website (listed below). Paired t-test was used to compare the TPM values in individual patients.

Syt1: https://www.proteinatlas.org/ENSG00000067715-SYT1/tissue/urinary+bladder

Syt2: https://www.proteinatlas.org/ENSG00000143858-SYT2/tissue/urinary+bladder

syntaxin-1: https://www.proteinatlas.org/ENSG00000106089-STX1A/tissue/urinary+bladder

synaptophysin: https://www.proteinatlas.org/ENSG00000102003-SYP/tissue/urinary+bladder

## Supporting information

S1 Fig Syt1^M3^ KI and Syt2^M3^ KI mice showed growth curves similar to their WT and heterozygous littermates. The body weights of Syt1^M3^ KI (TG/TG), Syt2^M3^ KI, WT, and heterozygous (WT/TG) male and female mice were recorded and plotted over time. n = 3–10 mice.
(TIF)

S2 Fig Syt1, but not Syt2, is detected in human bladder tissues. **A.** Immunohistochemistry analysis of human bladder sections, obtained from a commercial source with no underlying urologic pathology, showed Syt1 expression along nerve fibers (n.f.) within the detrusor (d.) layers, co-localized with synapsin. Syt2 expression is not detected. **B.** RNA sequencing of human post-mortem urinary bladder tissues showed higher levels of Syt1 transcripts than Syt2. pTPM: transcripts per kilobase million. Data obtained from Genotype-Tissue Expression (GTEx) project, accessed from www.proteinatlas.org. ****P<0.0001. **C.** The normalized and log_10_ transformed transcriptional read counts for synaptic vesicle proteins (Syt1, Syt2, SYN1, and SV2A) and bladder markers (NRP2 and UPK2) in mouse bladders are represented as a heatmap. The data were extracted from GEO (Genome Expression Omnibus) series GSE144295 and GSE149569. The normalization was performed in R (R Core Team, 2021) based on the total read count per sample. The heatmap was generated with heatmap2 function in the Morpheus R package (Broad Institute). Relative expression level was used for the color coding.
(TIF)

S3 Fig Evaluation of BoNT/B and BoNT/G using DAS assays on WT and Syt1^M3^ mice. **A.** The indicated doses of BoNT/B were used in DAS assays and the scores over time were plotted. **B.** The indicated doses of BoNT/G were used in DAS assays and the scores over time were plotted.
(TIF)

S4 Fig Syt1^M3^ bladder strips are less sensitive to BoNT/B than WT bladder strips. **A.** Contractile responses to electrical field stimulation of WT and KI mice, in the setting of high dose (1 nM) of BoNT/B. WT and Syt2^M3^ bladder strips have decreased contractile capability, while Syt1^M3^ bladders are largely resistant to the effect of BoNT/B. Only under long duration (300 mins) of 1 nM BoNT/B do Syt1^M3^ bladders begin to become slightly paralyzed. **B.** Carbachol treatment validates that bladder muscle viability and contractility remain unchanged after incubation with high dose BoNT/B. **C.** Survival curve for direct bladder wall injection of BoNT/B revealed the highest tolerable dose injected into the bladder is 3.25 pg. Systemic toxicity and death resulted from 4.25 pg bladder injection. WT 1 nM BoNT/B (n = 2); Syt1^M3^ 1 nM BoNT/B (n = 5); Syt2^M3^ 1 nM BoNT/B (n = 5).
(TIF)

S1 Table A list of antibodies utilized in this study. (DOCX)
