# Supplementary figures and images for: Knockin mouse models demonstrate differential contributions of synaptotagmin-1 and -2 as receptors for botulinum neurotoxins
Source: PLoS Pathog. 2021 Oct 18;17(10):e1009994. doi: 10.1371/journal.ppat.1009994 (PMC8553082; doi:10.1371/journal.ppat.1009994)

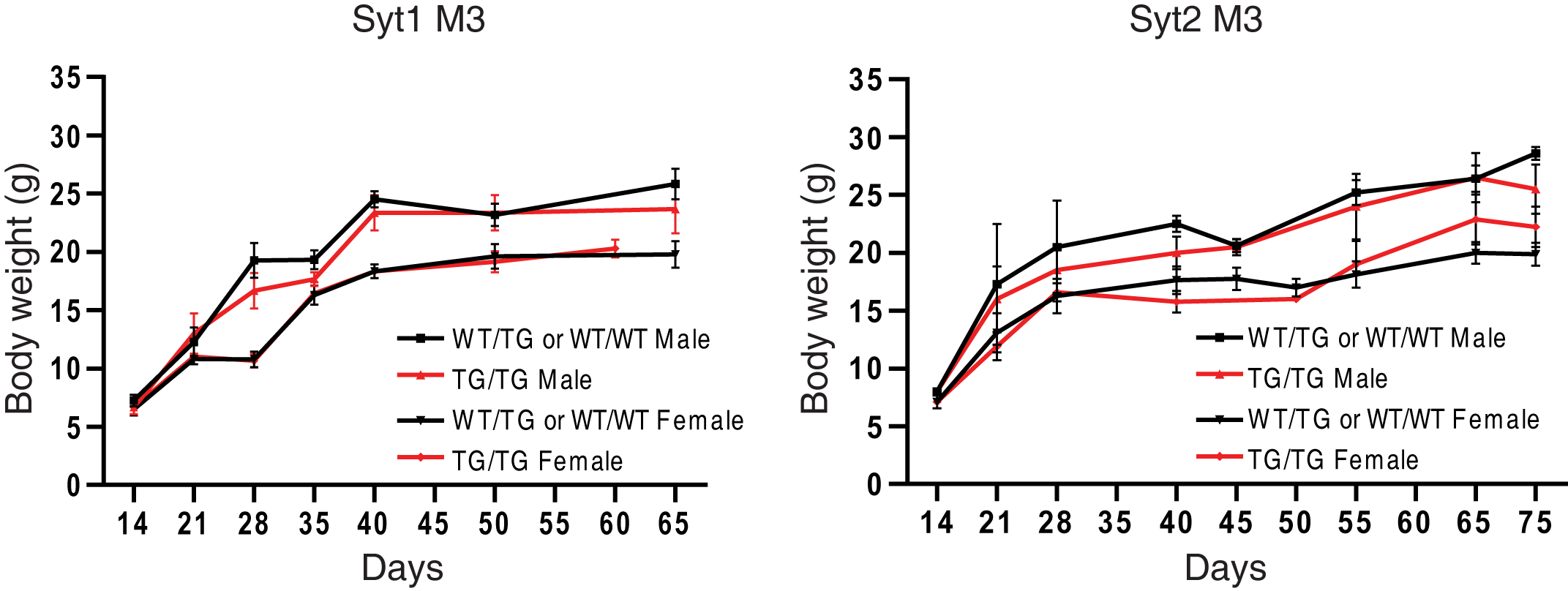

Supplement: S1 Fig — The body weights of Syt1M3 KI (TG/TG), Syt2M3 KI, WT, and heterozygous (WT/TG) male and female mice were recorded and plotted over time. n = 3–10 mice. (TIF) [file ppat.1009994.s001.tif]

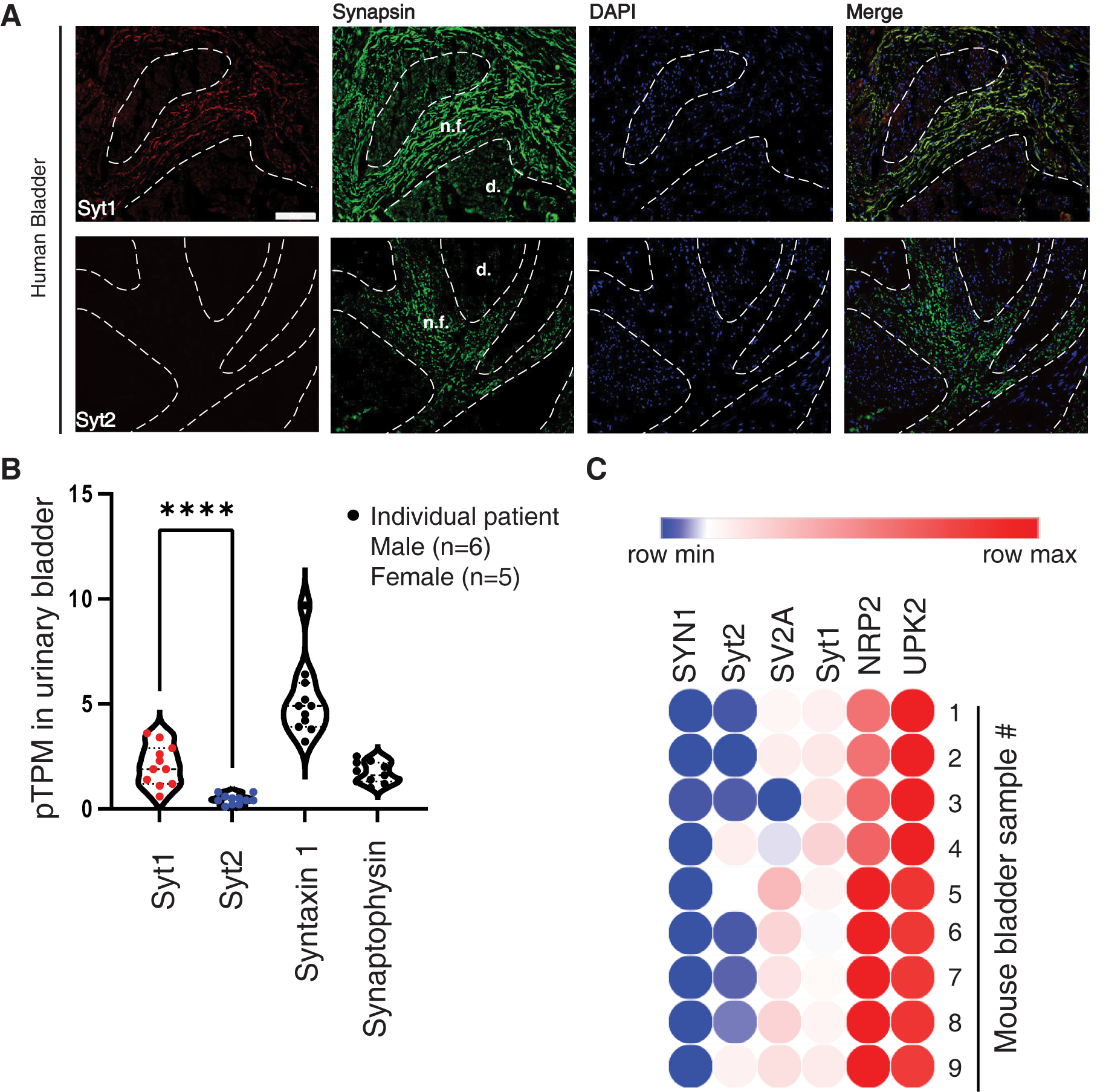

Supplement: S2 Fig — A. Immunohistochemistry analysis of human bladder sections, obtained from a commercial source with no underlying urologic pathology, showed Syt1 expression along nerve fibers (n.f.) within the detrusor (d.) layers, co-localized with synapsin. Syt2 expression is not detected. B. RNA sequencing of human post-mortem urinary bladder tissues showed higher levels of Syt1 transcripts than Syt2. pTPM: transcripts per kilobase million. Data obtained from Genotype-Tissue Expression (GTEx) project, accessed from www.proteinatlas.org. ****P<0.0001. C. The normalized and log10 transformed transcriptional read counts for synaptic vesicle proteins (Syt1, Syt2, SYN1, and SV2A) and bladder markers (NRP2 and UPK2) in mouse bladders are represented as a heatmap. The data were extracted from GEO (Genome Expression Omnibus) series GSE144295 and GSE149569. The normalization was performed in R (R Core Team, 2021) based on the total read count per sample. The heatmap was generated with heatmap2 function in the Morpheus R package (Broad Institute). Relative expression level was used for the color coding. (TIF) [file ppat.1009994.s002.tif]

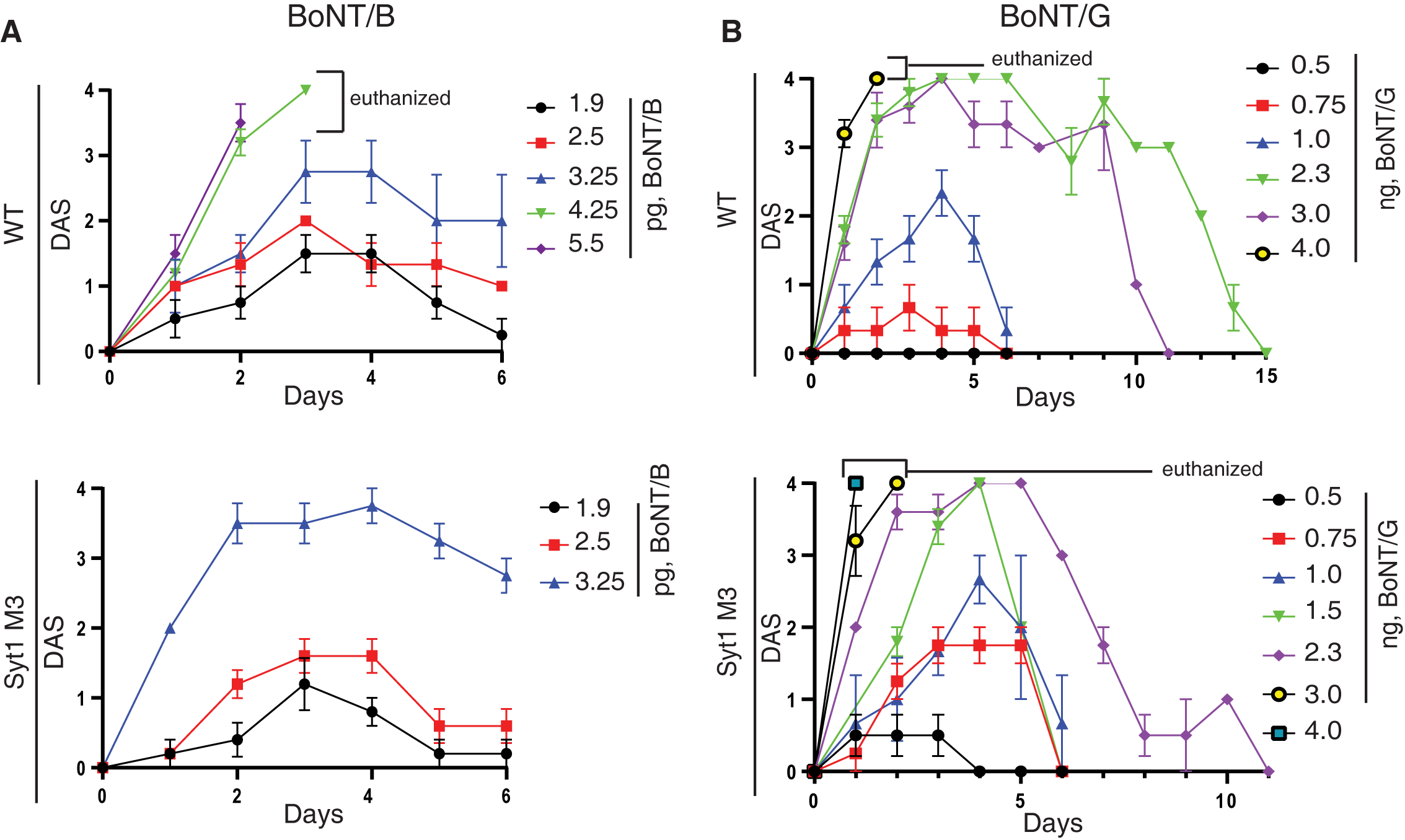

Supplement: S3 Fig — A. The indicated doses of BoNT/B were used in DAS assays and the scores over time were plotted. B. The indicated doses of BoNT/G were used in DAS assays and the scores over time were plotted. (TIF) [file ppat.1009994.s003.tif]

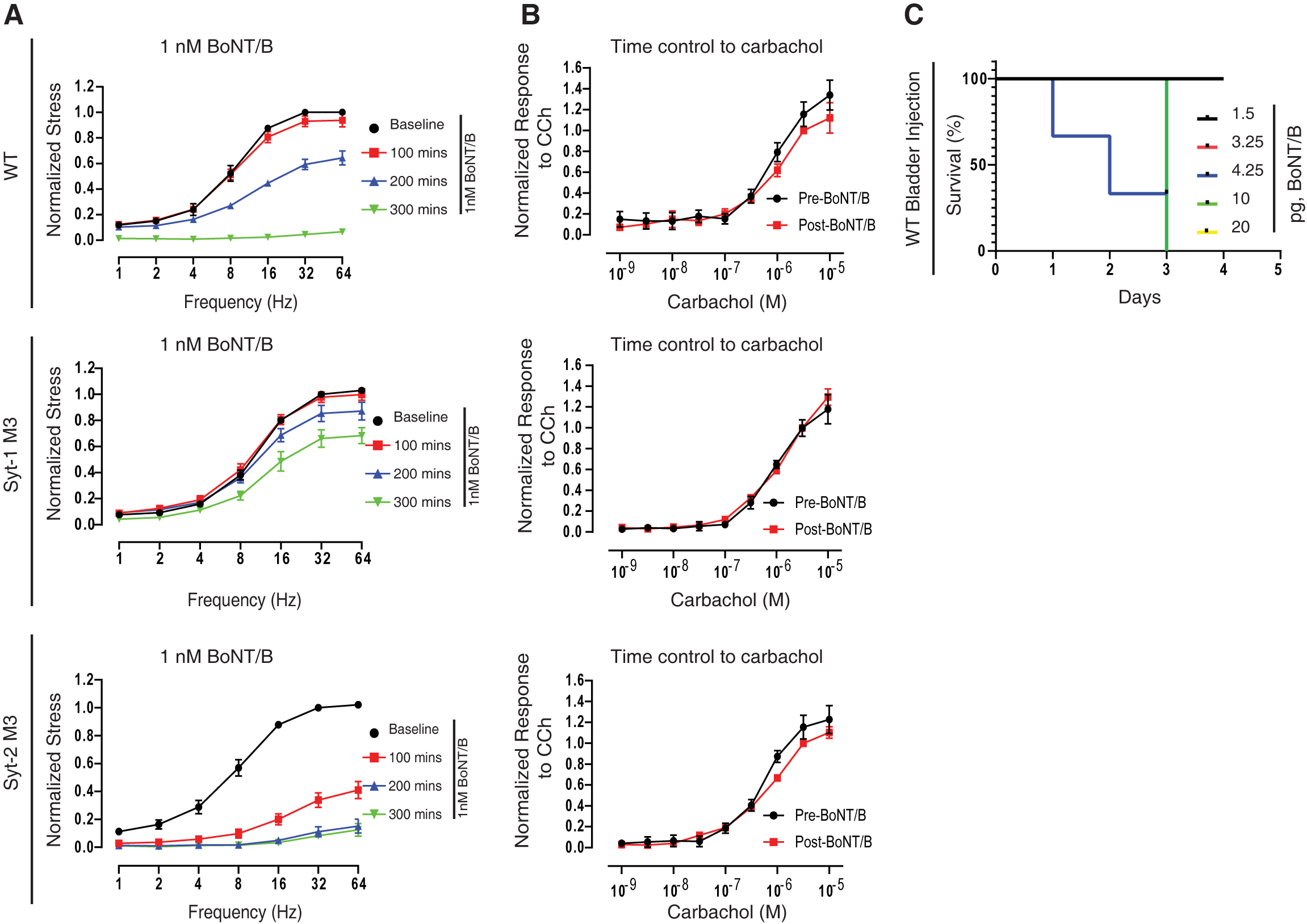

Supplement: S4 Fig — A. Contractile responses to electrical field stimulation of WT and KI mice, in the setting of high dose (1 nM) of BoNT/B. WT and Syt2M3 bladder strips have decreased contractile capability, while Syt1M3 bladders are largely resistant to the effect of BoNT/B. Only under long duration (300 mins) of 1 nM BoNT/B do Syt1M3 bladders begin to become slightly paralyzed. B. Carbachol treatment validates that bladder muscle viability and contractility remain unchanged after incubation with high dose BoNT/B. C. Survival curve for direct bladder wall injection of BoNT/B revealed the highest tolerable dose injected into the bladder is 3.25 pg. Systemic toxicity and death resulted from 4.25 pg bladder injection. WT 1 nM BoNT/B (n = 2); Syt1M3 1 nM BoNT/B (n = 5); Syt2M3 1 nM BoNT/B (n = 5). (TIF) [file ppat.1009994.s004.tif]
