# Supplementary material for: Knockin mouse models demonstrate differential contributions of synaptotagmin-1 and -2 as receptors for botulinum neurotoxins
Source: PLoS Pathog. 2021 Oct 18;17(10):e1009994. doi: 10.1371/journal.ppat.1009994 (PMC8553082; doi:10.1371/journal.ppat.1009994)
Supplement: S1 Table — (DOCX) [file ppat.1009994.s005.docx]

**S1 Table. A list of antibodies utilized in this study.**

| **Target** | **Tissue** | **Antibody reference** | **Provider** | **Dilution** |
| --- | --- | --- | --- | --- |
| β-3 tubulin | Bladder | 801211 | BioLegend | 1:500 |
| Synapsin | Bladder | Ab1543 | Abcam | 1:500 |
| Synaptophysin 7.2 | Bladder | Ab32127 | Abcam | 1:500 |
| Syt-1 | Bladder | Ab133856 | Abcam | 1:200 |
| Syt-2 | Bladder | BD 612716 | BD Biosciences | 1:200 |
| Anti-BoNT/B | Bladder | N/A | Metabiologics | 1:800 |
| α-bungarotoxin | Diaphragm | 13422 | Invitrogen | 1:1000 |
| SMI-312 | Diaphragm | 837904 | BioLegend | 1:400 |
| Syt-1 | Diaphragm | Ab133856 | Abcam | 1:200 |
| Syt-2 | Diaphragm | 105223 | Synaptic systems | 1:400 |
| Syt-1 | Human bladder | Ab133856 | Abcam | 1:200 |
| Syt-2 | Human bladder | BD 612716 | BD Biosciences | 1:200 |
| Synapsin | Human bladder | Ab1543 | Abcam | 1:500 |

| **Target** | **Tissue** | **Antibody reference** | **Provider** | **Dilution** |
| --- | --- | --- | --- | --- |
| Syt-1 | Brain | 105011 | Synaptic systems | 1:2000 |
| Syt-2 | Brain | BD 612716 | BD Biosciences | 1:2000 |
| Syntaxin | Brain | 110011 | Synaptic systems | 1:2000 |
| VAMP-2 | Brain | 104203 | Synaptic systems | 1:2000 |
| SV2 | Brain | N/A | Developmental Studies Hybridoma Bank | 1:2000 |
| SNAP-25 | Brain | Ab5666 | Abcam | 1:2000 |
| Actin | Brain | 60008-1 | Proteintech | 1:2000 |
